# Medico-Legal Inquiry

**Published:** 1860-12

**Authors:** 


Medico-Legal Inquiry, concerning the value of Testimony respecting facts as they appear to a Mind partly Conscious. By T. L. Wright, M. D., of Bellefontaine, Ohio.

This is a Pamphlet of 32 pages, re-printed from the Transactions of the Ohio State Medical Society. The object of the author is to show that the testimony of an individual in relation to facts supposed to have taken place, while such individual was partially unconscious from the influence of anæsthetics, is entirely unreliable. The subject is ably discussed by the author.

---

We have received a copy of "Hartshorne on Principles of Medicine," and also one of "Hodge on Diseases Peculiar to Females;" but we must defer a proper notice of them until we have had time to give them a more careful examination. They are for sale at the book-store of W. B. Keen & Co., on Lake street.

